# Intubation using VieScope vs. Video laryngoscopy in full personal protective equipment – a randomized, controlled simulation trial

**DOI:** 10.1186/s12871-021-01502-7

**Published:** 2021-11-22

**Authors:** Hannes Ecker, Simone Kolvenbach, Holger Herff, Wolfgang A. Wetsch

**Affiliations:** 1 Faculty of Medicine, University of Cologne, Kerpener Str. 62, 50937 Cologne, Germany; 2 Department of Anesthesiology and Intensive Care Medicine, University Hospital of Cologne, Kerpener Str. 62, 50937 Cologne, Germany

**Keywords:** Endotracheal intubation, Airway management, Difficult airway, Video-laryngoscopy, VieScope

## Abstract

**Background:**

VieScope is a new type of laryngoscope, with a straight, transparent and illuminated blade, allowing for direct line of sight towards the larynx. In addition, VieScope is disposed of after single patient use, which can avoid cross-contaminations of contagious material. This has gained importance especially when treating patients with highly contagious infectious diseases, such as during the SARS-CoV2 pandemic. In this context, VieScope has not been evaluated yet in a clinical study.

**Material and methods:**

This study compared intubation with VieScope to video-laryngoscopy (GlideScope) in normal and difficult airway in a standardized airway manikin in a randomized controlled simulation trial. Thirty-five medical specialists were asked to perform endotracheal intubation in full personal protective equipment (PPE). Primary endpoint was correct tube position. First-pass rate (i.e., success rate at the first attempt), time until intubation and time until first correct ventilation were registered as secondary endpoints.

**Results:**

For correct tracheal tube placement, there was no significant difference between VieScope and GlideScope in normal and difficult airway conditions. VieScope had over 91% fist-pass success rate in normal airway setting. VieScope had a comparable success rate to GlideScope in difficult airway, but had a significantly longer time until intubation and time until ventilation.

**Conclusion:**

VieScope and GlideScope had high success rates in normal as well as in difficult airway. There was no unrecognized esophageal intubation in either group. Overall time for intubation was longer in the VieScope group, though in an acceptable range given in literature.

Results from this simulation study suggest that VieScope may be an acceptable alternative for tracheal intubation in full PPE.

**Trial Registration:**

The study was registered at the German Clinical Trials Register www.drks.de (Registration date: 09/11/2020; TrialID: DRKS00023406).

## Background

The SARS-CoV-2 corona virus has been spreading around the world since November 2019 and was declared a pandemic by the WHO (World Health Organization) in March 2020. The primary infection vector is airborne, mainly through droplets and saliva. Approximately 6% of infected patients have to be admitted to an intensive care unit, many of which have to be endotracheally intubated in acute respiratory insufficiency [1–3] (https://www.awmf.org/uploads/tx_szleitlinien/001028l_S1_Atemwegsmanagement_2015-04-abgelaufen.pdf).

Due to the transmission route, the German Society for Anesthesia and Intensive Care Medicine (DGAI) recommends a high degree of caution when treating COVID-19 patients and “protective measures for medical staff that go beyond the previous hygienic measures” [4]. As endotracheal intubation is an aerosol-producing activity with the highest risk of infection, especially staff performing airway management is obliged to wear highest level personal protective equipment (PPE), consisting of a waterproof, long-sleeved gown, hood, gloves, protective goggles of face shield and a protective mask with particle filter (FFP2 / NIOSH N95 or higher) [4].

When intubation is necessary, one of the top priorities for self-protection of the personnel is to minimize exposure to aerosols. Therefore, ideally, a quick, safe intubation should be conducted, with a greater safety distance between the practitioner and patient than with conventional direct laryngoscopy. In order to facilitate this, it is recommended to perform video laryngoscopy, if possible [4].

Video laryngoscopes can transmit a video image obtained at the tip of the laryngoscope to an external monitor and thus provide an indirect visualization of the airway and intubation [5–8]. However, it is associated with higher cost intensity and a certain additional workload. Since video laryngoscopes are complex devices consisting of several parts, most of which are not single-use, special attention must be given to the appropriate disinfection of these devices. While some parts can be cleaned easily, others (like the monitors) are usually not fully resistant to liquids, cannot be immersed in disinfectant, and can only be cleaned to a very limited extent with aggressive disinfectants. A potential transfer of virulent material to the next patient (“cross contamination”) is a dreaded concern when using these devices under the mentioned conditions.

The VieScope (Androit Surgical LCCC, Oklahoma City, USA) represents a new type of laryngoscope, that is to be disposed of after single patient use, which can avoid cross-contaminations of contagious material.

We hypothesized that intubation success with VieScope would be comparable to video laryngoscopy in a contagious patient. Accordingly, the primary endpoint of this study was to compare success rates of endotracheal intubation (measured by verification of the correct tube position) with VieScope in comparison to a conventional video laryngoscope in a randomized, controlled simulation study. In addition, fist-pass-rate, time till intubation and time to first ventilation were also registered as secondary endpoints.

## Methods

### Ethics approval

The Ethics Committee of the University of Cologne approved the study on 26.10.2020 (ID 20-1465_1; Head: Prof. Dr. Drzezga).

Consent for study conduction during the pandemic was given from the hospital’s hygiene department, given that all participants were required to wear FFP2 / N95 respirators at all times.

### Study registration

The study was registered at the German Clinical Trials Register www.drks.de (Identifier: DRKS00023406) on 09.11.2020.

### Study design

This study was conducted from November 2nd to November the 6th 2020 in the facilities of the University Hospital of Cologne as a randomized controlled manikin trial.

Thirty-five physicians, who were all medical specialists from the Department for Anesthesiology and Intensive Care Medicine at the University Hospital of Cologne, volunteered to participate after giving written and informed consent.

Inclusion criteria were individuals working as physician in Anesthesia or Critical Care, and age between 18 and 65 years. Exclusion criteria were contraindication to wearing personal protective equipment and pregnancy or breastfeeding.

### Study protocol

After informed consent, the following demographic and medical background data of the test participants were recorded in pseudonymized form:

1. Gender
2. Age
3. Specialization
4. Medical experience level (years of professional experience)
5. Approximately how many intubations per year?

The participants were then asked to perform an endotracheal intubation on a certified airway training manikin (AirSim Advance X, TruCorp Ltd., Lurgan, Northern Ireland) in complete personal protective equipment (gown, hood, protective goggles, FFP2 respirator, gloves), using either video-laryngoscopy (GlideScope, Verathon Medical, Bothell, USA) or VieScope (Androit Surgical LCCC, Oklahoma City, USA) in randomized order.

Participants were asked to secure the airway of the manikin in four airway settings:

i) video laryngoscopy in normal airway
ii) video laryngoscopy difficult airway
iii) VieScope in normal airway
iv) VieScope difficult airway

In the scenarios with difficult airway, the tongue of the manikin was inflated to a pressure of 35 mbar to simulate a difficult airway situation.

The order of the scenarios was randomized using sealed opaque envelopes. A blocked randomization strategy was generated using an online tool (Sealed Envelope Ltd. 2020: www.sealedenvelope.com/simple-randomiser/v1/lists [Accessed 6 Oct 2020]).

Time measurements started with the beginning of airway measures (taking up the laryngoscope) and ended with the initial correct ventilation (using a resuscitation bag).

The following data was recorded for all four simulations and pseudonymized:

1. Tube position: tracheal vs. esophageal (primary endpoint)
2. Correct tube position at the first intubation attempt - “first pass-rate” (secondary endpoint)
3. Time until intubation (secondary endpoint)
4. Time until first correct ventilation (secondary endpoint)

Each simulation was terminated after successful intubation or after 5 min, at which irreversible hypoxia of the patient must be assumed.

### Materials

**Fig. 1:**
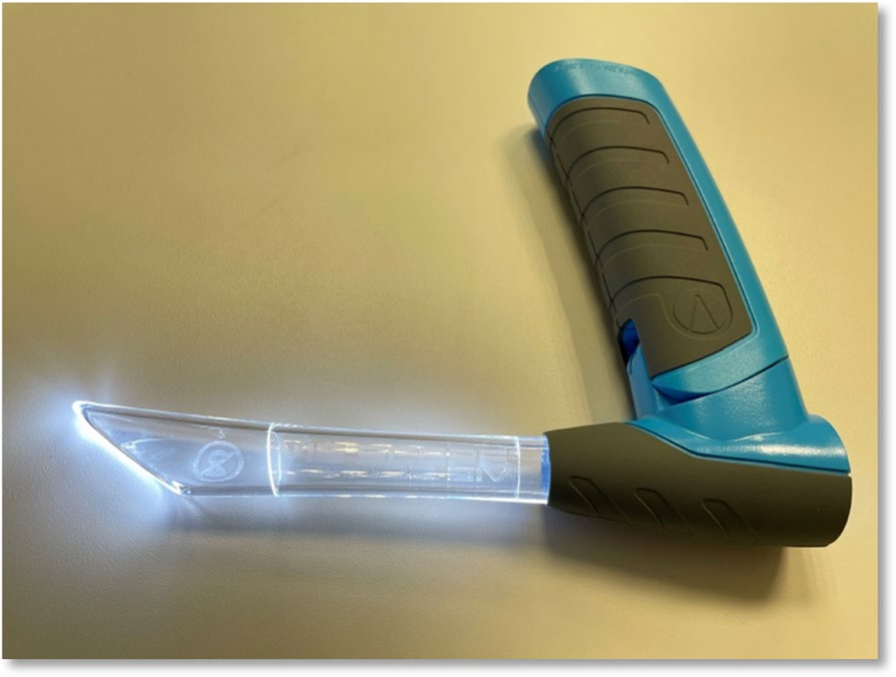
VieScope. VieScope, Adult Size (Androit Surgical LCCC, Oklahoma City, USA), in activated state with illuminated blade

**Fig. 2:**
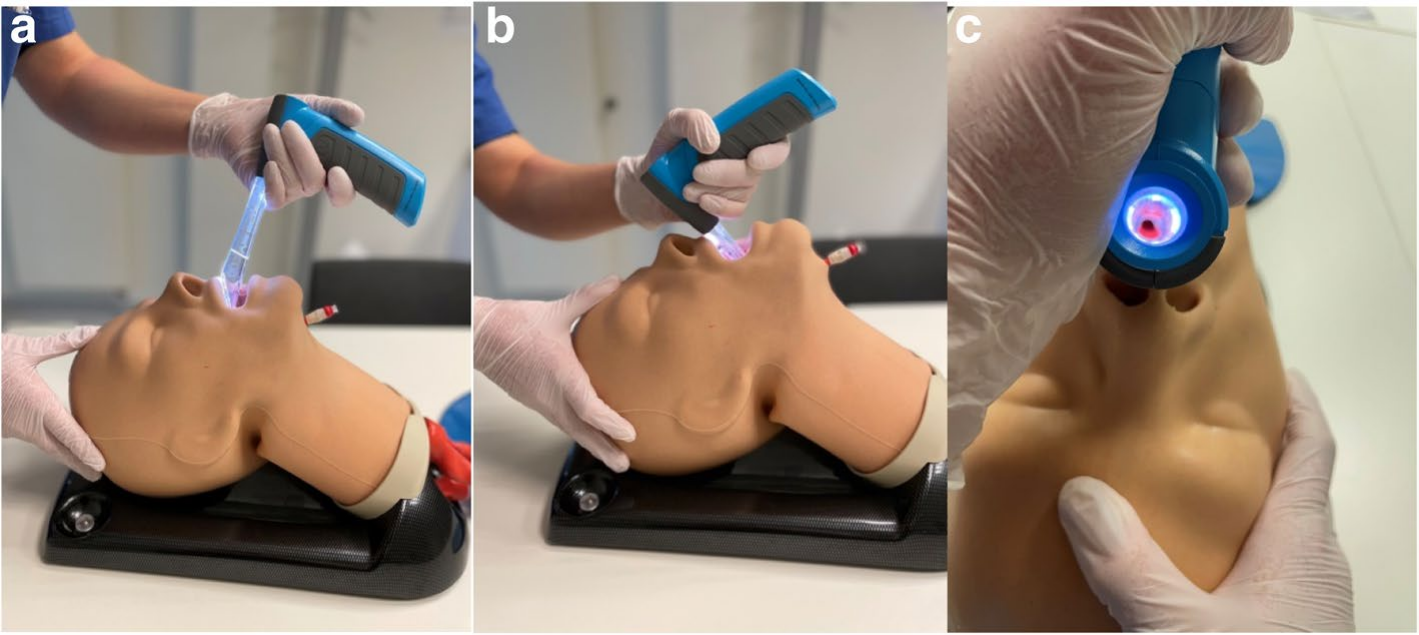
**a**-**c** VieScope deployment. VieScope insertion into a simulated adult airway (**a**-**b**). **c** shows visualization of the vocal cords for endotracheal bougie-placement.

For this study, the novel VieScope (VieScope “Training Demo”, Adult Size, Androit Surgical LCCC, Oklahoma City, USA; https://adroitsurgical.com/product/vie-scope) was used. It consists of a transparent circular straight tube (comparable to a Miller laryngoscope blade), which is illuminated by LEDs, and a battery handle. The VieScope is a standalone device, battery powered, and disposable after a single use (Fig. 1). As the scope itself has a straight, Miller-shaped blade, it facilitates a direct and straight view of the glottis, but it does not allow direct intubation. Instead, it requires the insertion of a bougie once sight to the vocal cords is achieved (Fig. 2a-c). Afterwards, an endotracheal tube can be passed into the trachea over the bougie, which then can be removed. As bougie, the VOIR Tactical Bougie (Androit Surgical LCCC, Oklahoma City, USA; https://adroitsurgical.com/product/voir-bougie) was used in the attempts with VieScope, whereas the rigid GlideRite stylet (Verathon Inc., Bothell, WA, USA; https://www.verathon.com/glidescope) was used for intubation with GlideScope (Verathon Inc., Bothell, WA, USA).

### Statistical analysis

Statistical computations were carried out using IBM SPSS Statistics (Version 25; IBM Inc., Armonk, NY, USA).

Sample size calculation revealed that 32 participants were required to detect a minimal difference of 20% difference with 80% power at a significance level of 5%. Thus, we decided to include 35 participants to account for possible dropouts.

For the comparison of the primary endpoint “Tube position” with GlideScope and VieScope in the different airway setting (“normal” and “difficult”), Fisher’s Exact Test was performed.

Secondary endpoints “first pass rate”, “time to intubation” and “time to ventilation”, were analyzed after normality test (Shapiro-Wilk) and equal variance test (Brown-Forsythe), using a one-way analysis of variance (ANOVA) for repeated measurements to determine the overall statistical significance between the groups. This was then followed by post hoc Student Newman Keuls method for pairwise multiple comparisons between two groups; *p* < 0.05 was considered as being significant.

## Results

### Demographic and background data

Thirty-five participants, all staff anesthesiologists or critical care specialists, were recruited for this study. Of these 35, 11 were female and 24 were male. The youngest participant was 26 years, the oldest 45 years old, while the average age was at 34 years. 40% (*n* = 14) had more than 6 years of experience in their field, while only 6% (*n* = 2) were in their specialty for less than a year. Ten participants (29%) specified that they perform 100–200 Intubations per year, while 15 (43%) performed over 200 Intubations annually.

All 35 (100%) had prior experience in the use of the GlideScope, while only 2 (6%) admitted experience in the use of the VieScope.

**Fig. 3:**
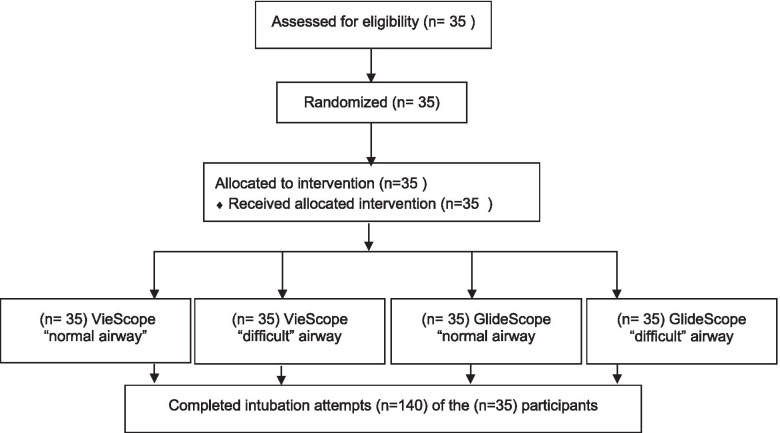
Flow chart. Each participant performed intubation in all settings in a randomized controlled order. There were no drop-outs

Each participant performed 4 endotracheal Intubation attempts in total, 2 with the GlideScope and VieScope, each on “normal” and “difficult” airways, in a randomized order. Thus, 140 data sets were acquired (Fig. 3).

### Tube position - endotracheal vs. esophageal

Regarding the primary endpoint of tube position in the first attempt in a normal airway setting, 35 (100%) had a correct endotracheal position with the GlideScope, while 32 (91%) had correct endotracheal position with the VieScope (*p* = 0.239).

In the difficult airway setting, the utilization of both GlideScope and VieScope resulted in 34 (97%) correct endotracheal positioning (*p* = 1.000).

### Correct tube position in first intubation attempt (first pass rate)

Tracheal intubation in normal airway using VieScope was successful at first attempt in 32 of 35 participants (91.4%), compared to 35 of 35 participants (100%) with GlideScope. In difficult airway, tracheal intubation with VieScope was successful at first attempt in 26 of 35 participants (74.3%), compared to 34 of 35 participants (97.1%) with GlideScope.

### Time to intubation

Regarding the secondary endpoint of time until successful endotracheal intubation in a normal airway setting showed a mean ± SD time of 21.7 ± 9.0 s for the use of the GlideScope versus 42.7 ± 35.9 s for the use of VieScope.

In the “difficult” airway setting, the mean time until successful intubation was 26.5 ± 20.5 s with the GlideScope, compared to 51.1 ± 30.3 s with the VieScope. An all pairwise multiple comparison procedures showed that the GlideScope had a significantly shorter time until intubation, compared to the VieScope in both the normal airway Setting (Difference of ranks 1436.500; q = 7.973; *p* < 0.001) and the difficult airway setting (Difference of ranks 1762.500; q = 9.782; p < 0.001).

### Time to first ventilation

Regarding the secondary endpoint of time until first ventilation in a normal airway setting showed a mean time of 33.1 ± 10.5 s for the use of GlideScope and 51.5 ± 37.7 s in VieScope group (Student-Newman-Keuls; *p* < 0.001).

In the “difficult” airway setting, the mean time until successful intubation was 36.6 ± 22.1 s with the GlideScope, compared to 61.4 ± 33.0 s with the VieScope (p < 0.001).

## Discussion

This is the first study that compares the novel VieScope to a form of video-laryngoscopy (GlideScope) for “normal” and “difficult airway” management in a randomized controlled simulation trial. Primary endpoint was “correct tube position”.

In addition, “first pass rate”, “time until intubation” and “time until first correct ventilation” were registered as secondary endpoints.

With regard to its primary endpoint “correct tracheal tube placement”, there was no significant difference between VieScope and GlideScope in “normal” and “difficult airway” conditions. Both devices had a high rate of success rates that are comparable or even higher than what can be expected from literature [9]. Especially in the difficult airway, both devices performed comparably well. We have no rationale why there was a difference between the two devices in the normal airway. Showing no significance, this may be a coincidence of represent that the participants were more experienced with GlideScope.

As for the secondary endpoints, VieScope had over 91% successful “fist pass rate” in a “normal airway” setting. In addition, it had a comparable success rate to the Glidescope in “difficult airway” setting. There were also no unrecognized esophageal intubations in both groups. Nevertheless, VieScope had significantly longer “time until intubation” and “time until ventilation” – duration, which may be relevant especially in a cohorte of COVID-19 patients, and which is not adequately represented in the primary study endpoint. Arguably, comparing intubation times is difficult, as the VieScope requires the insertion of a bougie, over which the endotracheal tube is placed, whereas the GlideScope is designed to allow direct endotracheal intubation – which bypasses one step. Different techniques of apneic oxygenation may prolong the time before desaturation occurs [10, 11]. However, this study showed that VieScope generally had acceptable Intubation times when comparing it to contemporary literature [9]. It may however be argued that – when timely intubation is extremely important (e.g., in patients that are already hypoxemic) – GlideScope may be advantageous in order to prevent the occurrence of severe hypoxemia during intubation. In addition, the mandatory use of a bougie for the VieScope increases the risk of hazardous intubation.

The VieScope itself was originally designed for deployment in EMS and combat medicine, and not truly for an in-hospital setting. However, its relatively small logistical effort, immanent “ready-for-use”-quality and “single-use” property, can make it an addition in in-hospital situations where other technologies are not, or only with delay, available. Combined with the fact that especially in airway management of patients with highly infectious conditions, such as those with SARS-CoV2, trying to avoid cross-contamination is obligatory, the device could be an alternative because of its properties.

The study was performed on a table on the same average height of an Intensive Care Unit (ICU)-bed. Still, intubation in a real ICU-bed can be even more challenging. Data from recent studies suggest that endotracheal intubation outside the operating room setting (e.g., on the ICU) is associated with increased difficulty and risks for the patients [12, 13]. Especially the typical problem of rapid deterioration of patients’ conditions cannot be adequately simulated in such a simulation study.

This study was conducted during the SARS-CoV-2 pandemic, at a time where no vaccines were available neither for patients nor for staff. Staff were obliged to wear a FFP2/N95 (or higher class) respirator all the time in hospital – which is something a healthcare worker was not used to before the pandemic. It would have been interesting to compare the performance of the two devices also when not wearing full PPE. Due to the pandemic situation, this was not possible at the time of the study, and may follow at a later time when it is safe to do this without risk for the participants.

### Limitations

Participating physicians in this study were all highly experienced in endotracheal intubation and the use of video laryngoscopy (GlideScope), since it is the primary and most-used and available tool in the participating hospital for difficult airway management. This may have given VieScope a relative disadvantage.

This study did not examine either whether the distance from patient to intubation-provider during airway management was larger in one of the groups or not, nor was it designed to account for aerosol and liquid spreading during this procedure. However, it should be noted that we observed a close distance between the physician’s face and the VieScope during the procedures. This prohibits a general safety recommendation of VieScope for these situations, without further research.

Lastly, manikin studies have the limitations of not being able to fully assess human factor elements (stress, cognitive overload etc.), as seen in real patients. However, the Trucorp manikin used in our study has been extensively evaluated and was found to be an alternative to the cadaver model for airway management studies [14]. In addition, in potentially contagious patients there is the fear of self-infection which further affects performance, especially during a difficult airway and cannot be fully assessed in a manikin study.

## Conclusion

VieScope and GlideScope had high success rates in normal as well as in difficult airway. There was no unrecognized esophageal intubation in either group. Overall time for intubation was longer in the VieScope group, though in an acceptable range given in literature. Results from this simulation study suggest that VieScope may be an acceptable alternative for tracheal intubation in full PPE.

## Data Availability

All data are included in the manuscript. The original datasets analysed during the current study are available from the corresponding author on reasonable request.

## Abbreviations

ANOVA: Analysis of variances
COVID-19: Coronavirus-2019 Disease
DGAI: German Society for Anesthesia and Intensive Care Medicine (DGAI)
EMS: Emergency Medical Services
FFP: Filtering Face Piece
ICU: Intensive Care Unit
LED: Light Emitting Diode
NIOSH: National Institute of Occupational Safety and Health
PPE: Personal Protective Equipment SARS-CoV2 Severe Acute Respiratory Syndrome- Coronavirus 2
WHO: World Health Organization
